# Modeling the occupational health risk of workers caused by environmental release during the production of PC components

**DOI:** 10.3389/fpubh.2022.1076461

**Published:** 2022-11-22

**Authors:** Peng Cui, Haifeng Zhao, Zhiyu Dong, Xuan Ju, Ping Zou, Siyu Zhou

**Affiliations:** School of Civil Engineering, Nanjing Forestry University, Nanjing, China

**Keywords:** prefabricate concrete, occupational health risks, environmental release, LCA, risk management assessment

## Abstract

Prefabricated construction is one of the solutions to the problem of balancing environmental improvements with the new buildings in the construction industry. Some work originally done on site is transferred to the front end, and the occupational health risks to industrial workers during the production of prefabricate concrete components are thus aggravated. This study aims to propose a framework to simulate the occupational health risks of workers in prefabricate concrete component plants from the perspective of risk identification, risk assessment, and risk control. Through the following 4 steps, including environmental release monitoring, diffusion and human inhalation mechanism analysis, occupational health risk evaluation, and full-path health risk simulation, this study maps physical entities to virtual reality. The proposed method tends to address the root causes behind occupational health risks, such as the lack of measurement, assessment and prevention criteria, and providing new ideas for theoretical research and innovative practice of HSE management and risk management in the construction industry.

## Introduction

The construction industry faces the problem of balancing economic growth and environmental impact. The concept of prefabricated building is an innovative solution to this problem. Compared to traditional forms of construction, prefabricated building has the advantages of increasing efficiency, shortening periods, lowering costs, saving energy, reducing consumption, reducing environmental pollution and sustainability in the production, construction and use processes (1, 2). Therefore, the prefabricated buildings are being promoted and used worldwide, which contribute to the construction industry's vision of a “carbon-free future.” Taking China as an example, the development of prefabricated buildings has been adopted as an important national strategy. The Chinese government began to focus on the promotion of prefabricated buildings with the *Green Building Action Plan* released in 2013. In 2018, the Chinese government further made the vigorous development of prefabricated buildings a key task and overall requirement for the future. In 2021, the Chinese government released the *Action Plan for Carbon Peaking by 2030*, which aims to accelerate the application of prefabricated buildings and the industrialization of construction. Even under the influence of the epidemic, the total building area of new prefabricated building in China reached 740 million square meters in 2021, accounting for about 25% of total area of new construction with more than 500,000 workers employed.

Prefabricated buildings can be divided into concrete structures, steel structures, bamboo and timber structures according to the type of structure, among which prefabricated internal/external wall panels, prefabricated floor slabs, prefabricated staircases and prefabricated balconies as the main components of prefabricated concrete structures are the most common, accounting for over 90% of the market share (3). Unlike traditional on-site construction methods, the production of prefabricate concrete components is mainly carried out in fully/semi-enclosed industrial plants. Industrial workers are required to put in intense physical work and are exposed to specific environments for long periods of time (4). During the production of prefabricate concrete components, the use of materials, machinery and energy leads to the generation of many types of harmful environmental release such as dust, noise, volatile organic compounds (VOCs) and radioactive elements (5).

The environmental protection authorities in most country will review the environmental assessment before the project goes into operation. However, the focus is on the impact of the plant's emissions on the external environment, while neglecting the occupational health of workers. For example, although a certain prefabricated concrete (PC) component manufacturer acquired ISO 9001 and ISO 14001 certification and meet the 17 elements of environmental management, there are only a few words of discussion concerning the occupational health of workers. In addition, through the preliminary site survey and visits, some PC component production enterprises, especially the small plants, have problems such as dust, noise and odor, and workers lack the necessary protective measures and awareness of health protection, which exposed them to serious occupational health risks. A series of occupational health-related laws and regulations are enacted worldwide, including the *Occupational Health and Safety Management System Guide* in the UK, the *Occupational Health and Safety Management System* in the US, the *Occupational Health Inspection Management Regulations* in China, the *Occupational Health and Safety Management System General Guide* in Australia, the *Occupational Health and Safety Management System Guidelines* in Japan and the international standard *Occupational Health and Safety Management System Requirements and Guidelines for Use*. However, the risk regulation system and standards for the assembly construction industry, which lies between manufacturing and construction industry, are still immature and require systematic theoretical research results to support the development of risk evaluation criteria and the control of the overall process.

Thus, this article is grounded in the intersection of engineering, environmental, and health management. A logical framework of occupational health risks was formed using risk management concept to identify the full pathway process of environmental release, combined with a personal exposure assessment method to form a simulation model of occupational health risk assessment for the whole process workers. It is also applied to real-life cases to form a complete set of long-term management models of occupational health risks caused by environmental release, providing a new paradigm for research in the field of occupational health risk management.

## Literature review

### Environmental release

The environmental release in this study refers to the hazardous substances or energy spilling, leaking, emitting or escaping into the external environment in physical or chemical form. The construction phase is long with many upstream and downstream industries, complex construction elements, and huge amounts of construction materials, machinery and energy, thus generating various types of environmental release at all stages of building production, transportation, construction, decoration and renovation (6). The main environmental release listed in order from the construction industry are: carbon dioxide (and other greenhouse gases), inorganic dust (minerals, metals, artificial dust), formaldehyde, noise, VOCs, high temperature and high humidity, organic dust, ammonia, radon, etc. A summary of the above environmental release sources and health impacts is shown in Table 1. According to the EU statistical report, more than 15% of construction workers deal with or contact dangerous substances for a long time, which induces various skin diseases; 32% of construction workers are exposed to fumes and vapors more than half of their working hours, causing respiratory diseases, silicosis, and even cancer (25). In 2019, 79,000 people in the UK construction sector suffered from occupational diseases. About 3,500 people died of cancer each year, and 5,900 people suffered from cancer (26). Based on the above facts, the incidence of occupational diseases among construction workers is 2–6 times higher than the average level of the whole industry, among which the health damage of workers caused by environmental release is particularly serious.

**Table 1:** Typical environmental release, sources and health impacts during construction.

**Environmental releases**	**Sources**	**Health impacts**	**References**
CO_2_	Building materials embodied carbon, fossil fuel combustion, and energy use	Causing greenhouse effect, destroying ecological environment, threatening human long-term health and survival	(7–9)
Inorganic dust	construction activities such as handling hoisting and installation works	Impairing Lung function, symptoms of cough, eye irritation, acute bronchitis, pulmonary edema and dyspnea. Entering the bloodstream can cause damage to the heart, liver and stomach	(10, 11)
Formaldehyde	Upholstery materials and adhesives	Suspected carcinogen, stimulating skin and mucous membrane, inhibiting cell function, destroy vision and retina. Causing abnormality of olfactory, lung function, liver and immune function	(12–15)
Noise	Using construction machinery and handling building materials	Causing hearing damage or loss, affecting emotional and mental health	(16–18)
VOCs	Artificial board, paint, coating, adhesive, carpet, wallpaper etc.	Some categories are carcinogen, causing irritation of eyes and respiratory tract, skin allergy, headache, sore throat and fatigue	(19, 20)
High temperature and humidity	Electric welding, maintenance, summer construction environment	Causing heat stroke disease, heat spasm and other heat stroke diseases, hypertension, endocrine disorders and other physiological functions abnormality	(21–24)

Traditional building products, especially concrete components, are mostly cast on site in an outdoor open environment, and the resulting harmful environmental release are easily dissipated into the atmosphere, so the exposure concentration/intensity of industrial workers is relatively low. The production of PC components is conducted in industrial plants, and the production process produces various harmful environmental release, such as dust, noise and VOCs. Industrial workers have long been in this semi-enclosed space with high environmental release and high intensity, suffering serious potential threat to the health in the short or long term.

### Occupational health risk

“Occupational health risk” refers to the possibility of work-related diseases or occupational diseases caused by workers' exposure to occupational hazard factors in the process of occupational activities, and its health damage consequences have specific probability and severity (27). Occupational health risks mainly come from the following three aspects: occupational diseases, safety accidents and mental health (28). Occupational health risk assessment is a comprehensive and system through the workplace occupational hazards identification and analysis of the specific application of risk assessment methods, assessment of laborers in the professional activities caused by exposure to occupational hazard factors in the process of the possibility of work related diseases or occupational disease, to predict its occupational health risk level, provide the basis for the corresponding risk control measures (29).

Occupational health risk assessment can be divided into qualitative, semi-quantitative and quantitative risk assessment methods. In the 1990's, European and American countries and international organizations successively issued occupational health risk assessment guidelines or norms to assess and manage the risk of hazardous substances in the workplace, For example: ICMM Operational Guidelines for Occupational Health Risk Assessment, Occupational Exposure Limits Evaluation Methodology, New Guidance on Inhalation Risk Assessment, Simple Elements of Chemical Occupational Hazard Classification and Control Techniques, Romanian Risk Assessment Methodology for Occupational Accidents and Occupational Diseases, Australian Guidance on Occupational Health and Safety Risk Assessment Management, International Council on Mining and Metals Occupational Health Risk Assessment Guidance, Risk Assessment Methodology for Occupational Exposure to Toxic Chemicals, ICI Mond Toxicity Index Evaluation Method and so on (30–33). In addition to these classical models, Monte Carlo simulation, exposure proportional assessment method, integrated index assessment method, Physiologically-based pharmacokinetic (PBPK) model, fuzzy Bayesian network, and risk assessment method under operational conditions are also widely used (34–40).

The United States National Institute for Occupational Safety and Health (NOSH) research report indicated that there are as many as 39 categories related to occupational health and safety in the construction industry, such as: sandblasting, blood lead, asbestos, asphalt, carbon monoxide, eye diseases, high temperature and pressure, quartz, skin exposure, noise and so on. At present, in addition to Australia, Sweden, Germany and other countries with good occupational health management, there are still some countries in the world with relatively weak research on occupational health management in the construction industry, and there is still a great room for improvement in occupational health management in the construction industry.

Previous research OHSAS and HSE management system as the core, aiming at comprehensive evaluation of the construction industry practitioners of occupational safety and health risks. In fact, multi-source harmful environmental release is one of the causes of occupational health risks. From the perspective of building types, studies mainly focus on traditional building forms and their construction processes, while studies on prefabricated buildings, especially the PC component production are relatively few. In terms of the type of emissions, the vast majority of studies focus on emissions of carbon dioxide and other greenhouse gases that have profound effects on human health and the environment. However, there are few studies on environmental release such as dust, noise and VOCs that pose acute/chronic threats to the health of construction workers. In addition, previous studies lack real-time and long-term monitoring and dynamic optimization of emission sources and their surrounding environment, resulting in the lack of universality of static data obtained from a single measure, and the lack of referential results and conclusions.

## Materials and methods

### Life cycle risk management evaluation

**Figure 1:**
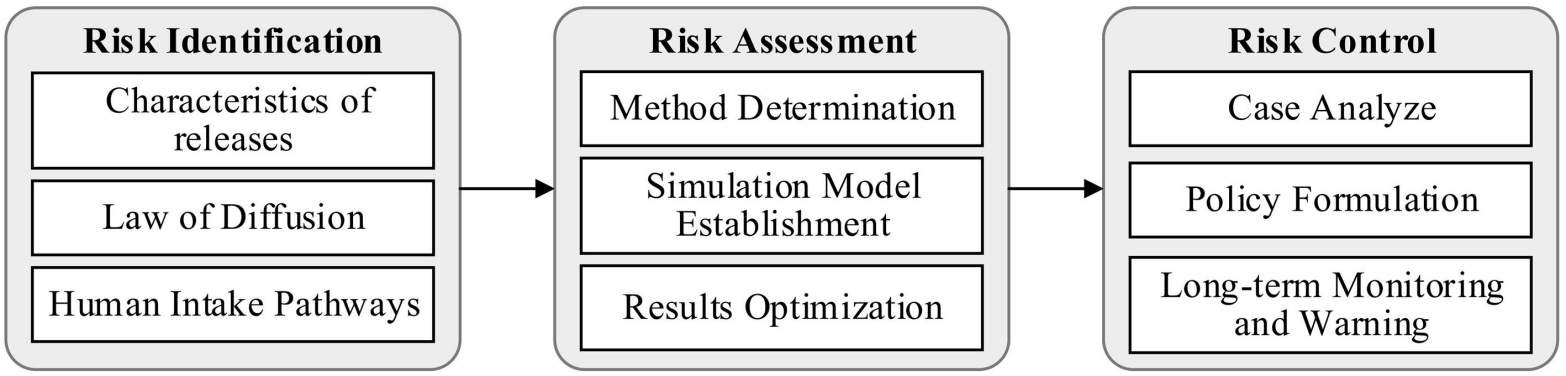
Life cycle risk management evaluation theory concept process.

The Life Cycle Assessment (LCA) can be used to assess the impact on human health of various pollutants emitted from the entire process of product production, with emphasis on a long term and cyclical evaluation concept (41). Risk Management Theory is to maintain a certain state of affairs when risk is deemed acceptable and to ensure maximum return. And when the risk is determined to be unacceptable, corresponding measures are taken to reduce the risk. Based on the above two theories, a life cycle risk management evaluation model is proposed in this paper to establish a risk management evaluation system as a long-term effect. The conceptual process is shown in Figure 1.

The risk identification is divided into three parts through the investigation and literature review to understand the mechanism of environmental release from the perspective of LCA. In the Risk Assessment stage, the occupational health risk assessment method and a simulation model adapted to the PC component production phase are built, so that the risks can be can be quantified and optimized. Finally, the modeled risk assessment system is put into empirical analysis. We will control the risk status of all aspects accurately and put forward policy advice. With the records of the database, the effect of measures on risk reduction are tracked and monitored, and the information is feedbacked to the risk evaluation and risk management system to achieve dynamic risk control (42).

### Occupational health risk assessment method

Given that the uncertain factors occur as probabilistic phenomenon, the individual exposure assessment method is used in this study. The individual exposure assessment method is used to simulate the probability of inhalation, exposure and exposure to hazardous environmental release during a worker's long-term work, and thus to assess occupational health risks (43). It can be calculated after a large number of iterations to make the results true and reliable within a certain confidence interval. According to the US EPA risk assessment model, the relationship between pollutant concentrations emitted by production and the intake dose of workers is calculated as Equation (1) (44).


(1) $$\begin{array}{l} CDI_{\text{i}}=\frac{C_{i}\times IR\times ED\times EF\times EL}{BW\times AL} \end{array}$$

Where *CDI*_i_ denotes the chronic daily inhalation/intake of emission i (mg/kg/d), *C*_i_ is the concentration of emission i (mg/m3), *IR* is inhalation rate (m3/h), *ED* represents exposure duration (h/d), *EF* is exposure frequency (d/a), *EL* is years of exposure, *BW* is body weight (kg), and *AL* is average life cycle (years).

In the available health risk assessment literature, hazardous compounds are usually classified as non-carcinogens or carcinogens (45). For carcinogenic substances, the carcinogenic damage quantification method is established the carcinogenic risk model based on the given carcinogenic slope factor and reference concentration data (46); for non-carcinogenic substances, a chronic disease damage quantification method is established for workers' occupational health risks by calculating chronic hazard factors based on reference concentration data (47). The carcinogenic risk model and the chronicity risk model of the occupational health risk model were quantified using Equations (2) and (3), respectively (48).


(2) $$\begin{array}{l} CR_{\text{i}}\;=\;CDI_{i}\times ISF_{i} \end{array}$$

(3) $$\begin{array}{l} HQ_{i}\;\text{=}\;\frac{C_{\text{i}}}{RfC_{i}} \end{array}$$

Where *CR*_i_ denotes carcinogenic risk of emission i, *ISF*_i_ is inhalation slope factor for emission i (kg·d /mg), *HQ*_i_ means Hazard Quotient, namely the ratio of the concentration of emission i to its reference concentration *RfC*_i_ in a given time period, with *HQ*_i_ ≥1 indicates that the emission concentration is high enough to cause chronic non-carcinogenic effects. *RfC*_i_ symbolizes the reference concentration factor of emission i (mg/m3). *ISF*_i_ and *RfC*_i_ are captured in the US Integrated Risk Information System (IRIS).

The disability-adjusted life year (*DALY*) is used to quantify the disease burden and injuries in human populations in the Global Burden of Disease Study and perform a quantitative assessment of health damage (49). Health risks are proportionally distributed with the diseases suffered through impact and damage analysis and unified into the *DALY* as shown in Equation (4) (50).


(4) $$\begin{array}{l} DALY=YLL+YLD \end{array}$$

Where *YLL* denotes the year of life lost. *YLD* is the year of life lived with a disability. It is a time-based measure that combines the time lost due to premature mortality and the duration of disability caused by illness in survivors.

## Results

**Figure 2:**
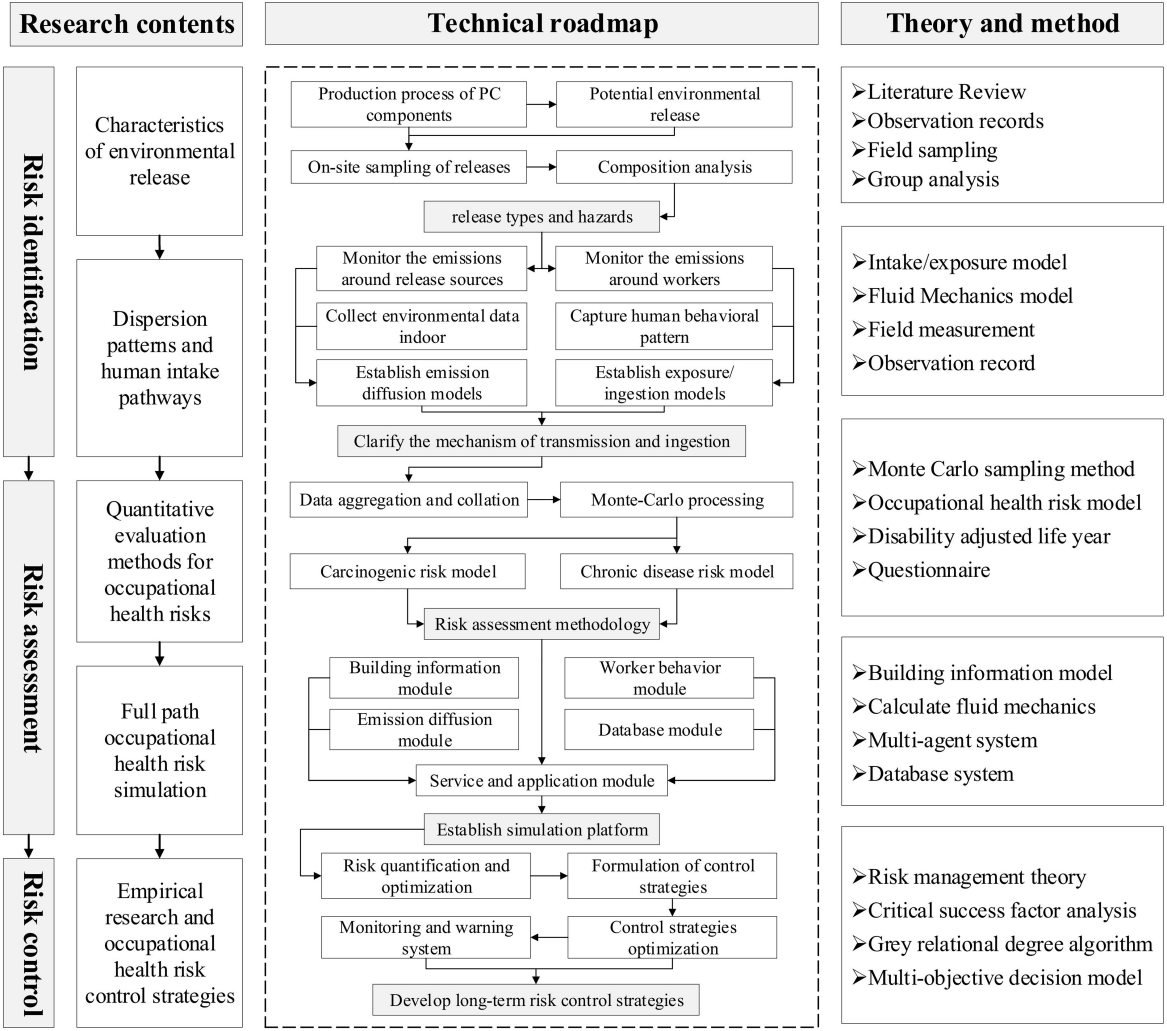
Occupational health risk framework.

According to the three stages of risk management theory, this study builds an occupational health risk framework, starting from the three stages of risk identification, risk assessment and risk control, including research content, technical roadmap and methods, as shown in Figure 2.

### Risk identification

**Figure 3:**
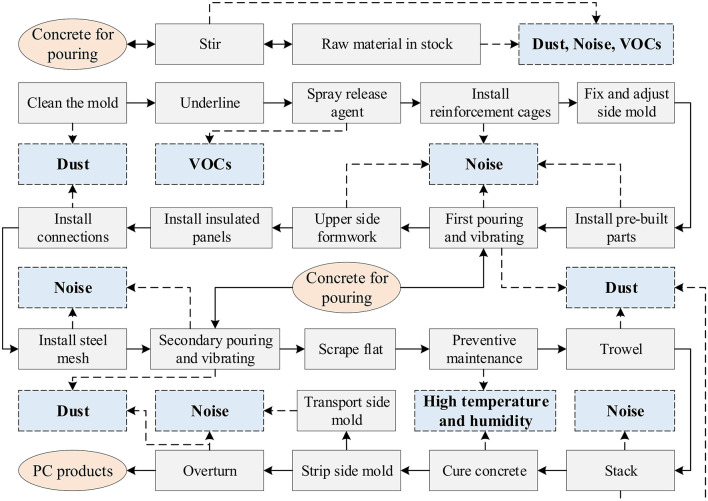
Types of potentially harmful environmental release from the production of precast concrete facade panels.

Identifying the source of environmental release and confirming the emission characteristics. Specifically, it includes the following two contents: (1) Sorting out the current production process and process characteristics of typical prefabricate concrete component plants assembly lines. (2) Determining the type, location and extent of environmental release resulting from operations such as material fugitive, energy consumption and mechanical equipment operation during the production of prefabricate concrete components. Taking PC exterior wall panels as an example, their production contains more than 30 processes such as cleaning the mold, installing reinforcement cages, pouring and vibrating, and spray release agent, etc. The potential harmful environmental release during the process mainly contains dust, noise, and VOCs, as shown in Figure 3. For other types of PC components, such as PC floor slabs, PC staircases, PC balcony slabs required for housing construction, and PC shield pieces required for underground projects, are slightly different in process, but the main types of environmental release are the same.

Detection and analysis of environmental release properties in precast concrete components plants includes: (1) Field sampling of dust, VOCs gas and other aerosol substances using cyclone particulate collectors, vacuum sampling pumps, Teflon filter membranes, etc. Samples collected in the field are sent to the laboratory under light-free, low-temperature and dry conditions. (2) The material class and particle size interval of the solids in the filter membrane, and the composition and proportion of the gas in the sampler, respectively, with the aid of gas chromatography/mass spectrometry (GC/MS), according to the standard test method provided by the National Institute for Occupational Safety and Health Research (NIOSH). (3) Toxicological and human potential damage analysis was conducted for all detected substances, and the substances with a large proportion and heavy hazard were finally selected as the final environmental release types (Note: Noise is analyzed separately for its impact on human health and is included as one of the environmental release types).

### Risk assessment

Based on the source emission characteristics, the diffusion and dispersion mechanisms of environmental release are analyzed using spread and dispersion laws as follows: (1) Using monitoring equipment such as laser dust meter, acoustic meter and photo-ionization detector to monitor the emission sources and the surrounding environment at fixed points to obtain real-time data on the change in concentration of dust or gas emissions during the production of prefabricate concrete components and the change in intensity of noise, respectively. (2) Collecting and measuring data on potential influencing factors such as spatial boundaries, wind speed, wind direction, temperature and humidity within the plant during the same production time period using temperature/humidity meters, anemometers and so on. (3) Establish an environmental release propagation/diffusion model, and simulate the transport trajectory, diffusion path and concentration distribution of dust and gas, as well as the propagation distance and decreasing degree of noise, respectively, with Fluent software. (4) Determine the propagation or dispersion pattern of various types of environmental release during the production of prefabricate concrete components.

**Figure 4:**
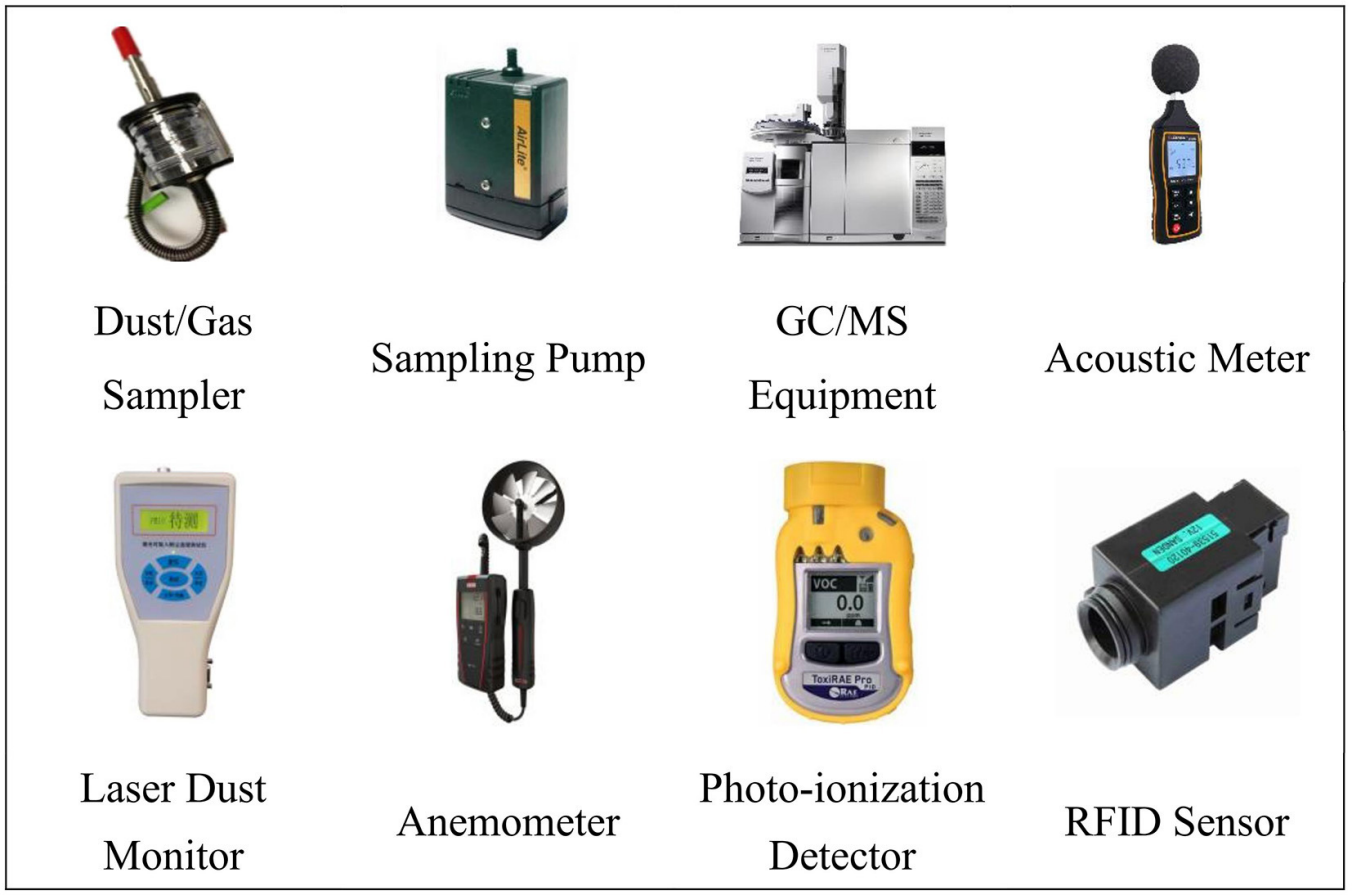
Main instruments and equipment proposed for use.

The human intake mechanism of environmental release includes the following four steps: (1) Real-time monitoring of noise intensity and emission concentration data at the ear, mouth and nose of workers of different job types during production operations by requiring workers to wear portable devices. (2) Deploy RFID sensor devices at designated locations to collect and record data on workers' job characteristics, behavioral habits, movement range and other potential influencing factors. (3) Modeling the inhalation/exposure dose of workers to simulate their inhalation, exposure dose, or exposure level to short-lived environmental release under specific constraints. (4) Determine the way of environmental release from human intake during the production of prefabricate concrete components. A number of instruments and equipment are used in these processes, which are highly specialized and sophisticated. The main instruments and equipment mentioned above are shown in Figure 4.

### Risk control

**Figure 5:**
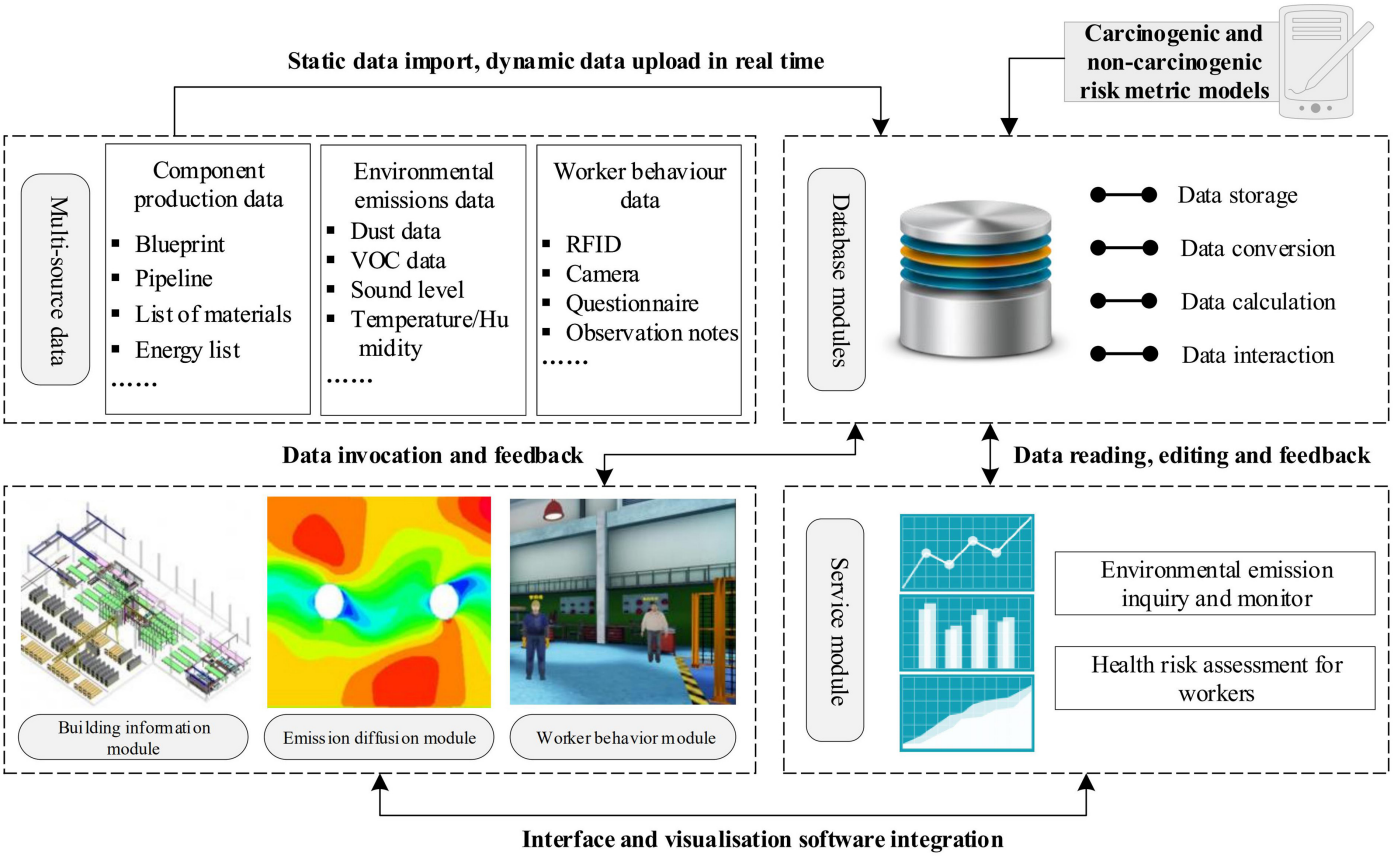
Simulation and evaluation model for occupational health risk assessment.

On the basis of the risk identification and assessment methods, the simulation model was established to establish the “Emission-Transmission-Inhalation” whole process of worker occupational health risk simulation and evaluation model. The simulation module of each part is established, specifically including: (1) Establish a basic simulation module of prefabricate concrete component production process and emission source location based on building information model. (2) Establish a numerical simulation module based on computational fluid dynamics to simulate the propagation and dispersion of environmental release in component plants. (3) Establish a multi-intelligence-based worker production behavior and health injury simulation module. (4) Create a multiple sources database module containing information on environmental release monitoring, worker health data, emission factors and so on. (5) The application development platform is selected to integrate the above simulation modules and database to build a quarriable, editable and calculable occupational health risk simulation and evaluation model, as shown in Figure 5.

Based on the life cycle risk management evaluation method, combined with the above simulation model, the long-term control of occupational health risks caused by environmental release is formed. For the identification of risk control strategies, the target case analysis takes into account the results of risk evaluation and identifies control strategies based on the classification of carcinogenic risk and chronic disease risk respectively. Then, based on the Critical success factors (CSF) analysis method, the key success variables of environmental release risk control are searched from three aspects: emission sources, transmission paths and recipient individuals.

**Figure 6:**
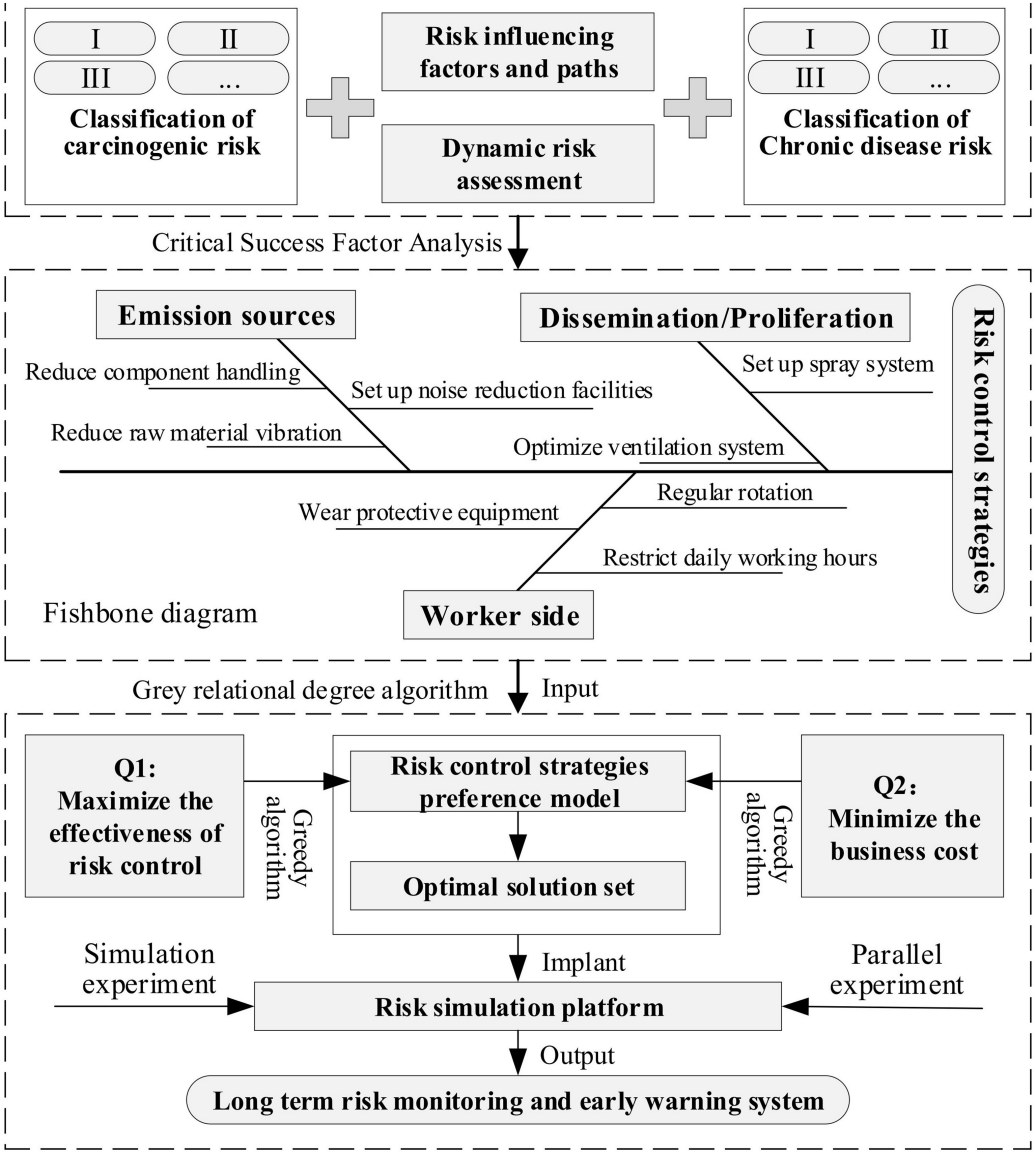
Long-term control model for occupational health risks caused by environmental release.

Drawing a fishbone diagram to identify, define and develop specific measures included in the above three CSFs, such as reducing dust/gas release due to handling, disturbance or vibration of parts, installing sound insulation and noise reduction equipment, installing sprinkler systems, optimizing ventilation systems, temperature and humidity regulation systems, regular cleaning and decontamination, requiring workers of specific jobs to wear protective equipment, strictly setting daily/yearly working hours for workers of certain special jobs, adopting a job rotation system on a regular basis, etc. Fishbone diagram through critical success factor analysis, the gray correlation algorithm is used to determine the gray correlation between the above measures and to develop the optimal combination strategy. The process is shown in Figure 6.

## Discussion

### Thoughts on the building of occupational health risk assessment model

The simulation model contains five key elements as shown in Equation (5), where: *SM* is the simulation model, *PE* is physical entity, *VE* is virtual entity, *SS* is service, *DB* is data, and *CN* is the connection between the parts.


(5) SM=(PE,VE,SS,DB,CN)

Physical layer element identification and data acquisition. The accurate analysis of *PE* is the basis for building the simulation model. In the project, a single PC component production machinery and equipment can be regarded as a unit-level *PE*, which is the smallest unit for function realization; a full set of PC component production line can be regarded as a system-level *PE*, which can complete the component production task; the whole plant composed of production line, environmental Emission and workers can be regarded as a system-level *PE*, which is a comprehensive system including material flow, emission flow and information flow, including four parts of human-machine-material-environment, as shown in Equation (6).


(6) $$\begin{array}{l} PE=\left(Hp,Mp,Pp,Ep\right) \end{array}$$

*Hp* describes the scope of action and behavior of industrial workers in the production process of components, and the data are obtained through RFID and image recognition technologies; *Mp* describes the operation of production machinery and production lines, and is portrayed and simulated through preliminary research and continuous probability events; *Pp* describes the production process of PC components, and is built through the material flow model; *Ep* describes the intensity and propagation law of environmental release, and is measured through sensors and portable devices.

Model layer model building and rules making. The establishment of the model layer is the core of the simulation technology. The digital model of this project includes geometric model, behavioral model and rule model, which describes and portrays each physical entity in the production process of PC components from multiple time scales and multiple space scales, as shown in Equation (7).


(7) $$\begin{array}{l} VE=\left(G\text{v},Bv,Rv\right) \end{array}$$

*Gv* is a 3D model describing the geometric parameters and relationships of *PE*, with good spatial and temporal consistency with PE, which can be realized by Fluent 3D modeling software with Revit and Auto CAD; *Bv* describes the real-time response and behavior of *PE* under the joint action of external environmental impact and internal operation mechanism in different time scales, using a multi-intelligence-based simulation model. *Rv* describes how physical entities operate, including rules based on historical data, experience based on tacit knowledge summaries, and process flow standards, enabling *VE* to map the PC component production process and environmental release in real time.

Functional and business module design of the service layer. The service layer is designed to encapsulate various types of data, models, algorithms, and results in the simulation model in a service-oriented manner, to support the operation of the internal functions of the model and to provide functional services (*Bs*). At the same time, the service (*Fs*) needs of PC component manufacturing enterprise managers for environmental release and worker health risk information acquisition are met through application software and clients, as shown in equation (8).


(8) $$\begin{array}{l} SS=\left(Bs,Fs\right) \end{array}$$

Fs includes: model management services provided for *VE* such as modeling simulation, model assembly and fusion; data management and processing services provided for *DB* such as data storage, encapsulation, cleaning, correlation and fusion; comprehensive connection services provided for *CN* such as data collection, sense access, data transmission, protocols and interfaces, etc. *Bs* mainly include: environmental release monitoring and occupational health risk assessment services for management personnel and other services; abnormal concentration monitoring and alarm services for industrial workers; data statistics and industry standard cap monitoring for government regulatory departments.

Database establishment and data are interconnected in the data layer. The establishment of the database in the data layer is the key to realize the information linkage and transmission among other layers, mainly including *PE* data, *VE* data and *SS* data, as shown in Equation (9).


(9) $$\begin{array}{l} DB=\left(D\text{p},Dv,Ds\right) \end{array}$$

*Dp* mainly includes physical element attribute data reflecting *PE* specifications, functions, performance, relationships, etc., as well as dynamic process data reflecting *PE* operating conditions, real-time performance, environmental parameters, sudden disturbances, etc., which can be collected through testing equipment, sensors and other equipment; *Dv* mainly includes data related to geometric models, physical models, behavioral models and rule models in *VE*, as well as simulation data based on the above models *Ds* mainly includes data related to *Fs* (such as algorithms, models, data processing methods, etc.) and *Bs* (query, analysis and management related data).

Inter-layer connection method design. After the above four layers are established, how to connect the layers together is another key technique to realize the proper operation of the simulation model. The connection (*CN*) between the above four layers is shown in Equation (10).


(10) $$\begin{array}{l} CN=\left(PD,PV,PS,VD,VS,SD\right) \end{array}$$

**Figure 7:**
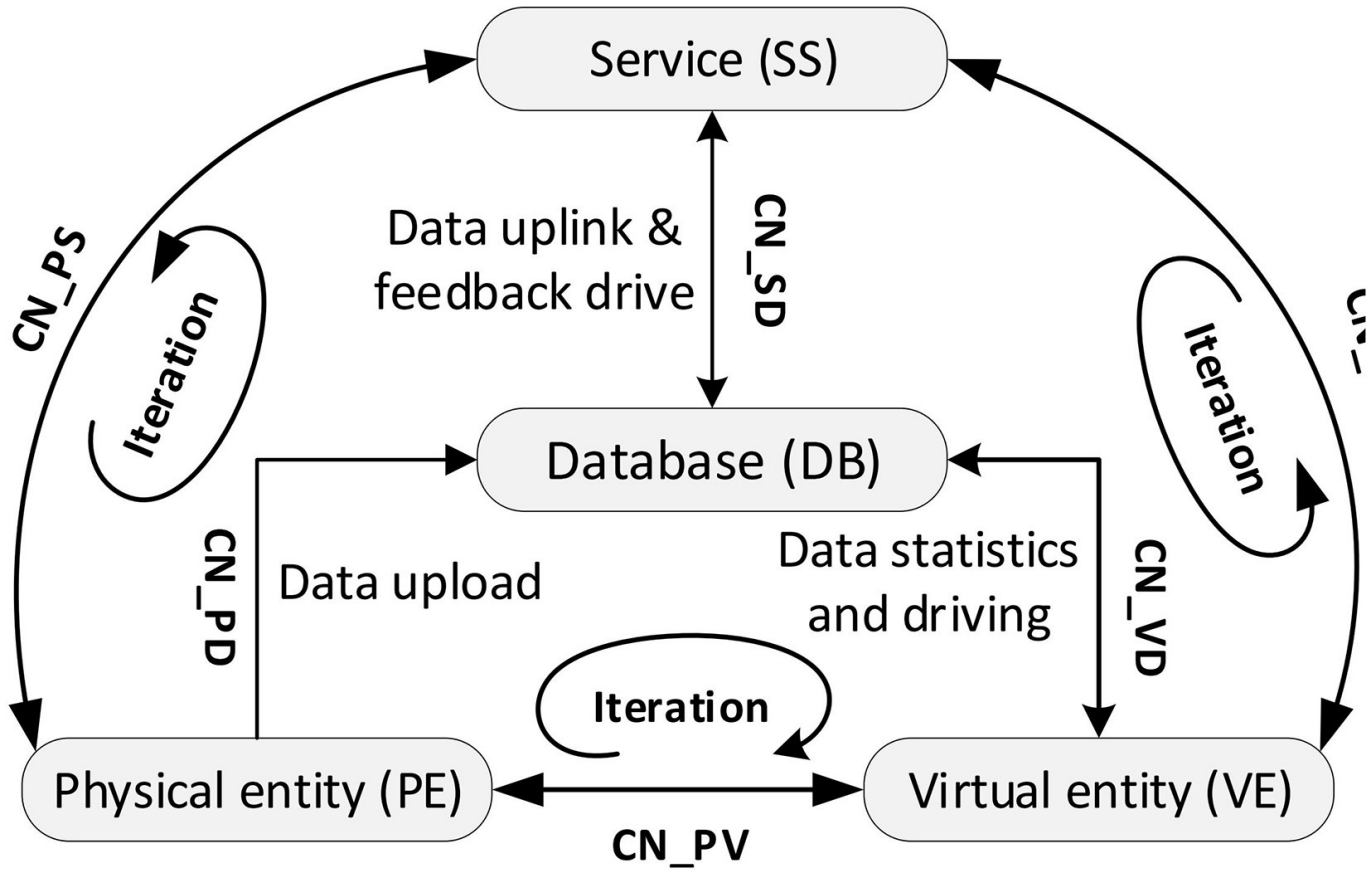
Model driven architecture of occupational health risk assessment.

*PD* realizes the interaction between *PE* and *DB*, using emission monitoring instruments, FRID, image recognition, etc. to collect *PE* data in real time and transmit them to *DB* through OPC-UA protocol specification; *PV* realizes the interaction between *PE* and *VE*, transmitting the collected *PE* real time data to *VE* for updating and correcting the model; *PS* realizes the interaction between *PE* and *SS*, transmitting the collected *PE* real time data to *SS* for updating and correcting the model. Realize the update and optimization of *SS*. At the same time, the operation guidance, analysis and decision optimization results generated by *SS* are provided to the manager in the form of client, and the optimization of *PE* is realized through manual operation; *VD* realizes the interaction between *VE* and *DB*, and stores the simulation and related data generated by *VE* into *DB* in real time through the database interface, and reads the associated data from *DB* in real time; *VS* realizes the interaction between *VE* and *SS*, and completes the instruction *VS* implements the interaction between *VE* and *SS*, completing commands such as instruction transmission, data sending and receiving, message synchronization, etc.; *SD* implements the interaction between *SS* and *DB*, storing *SS* data to *DB* in real time, and reading historical data and rule data in *DB* in real time. The three processes, *PV* in implementing the interaction between *PE* and *VE, PS* in implementing the interaction between *PE* and *SS*, and *VS* in implementing the interaction between *VE* and *SS*, are repeated over and over again, both iteratively, and the result of each iteration is used as the initial value for the next iteration. The model driven architecture of occupational health risk assessment is shown in Figure 7.

### Risk management strategy optimization and policy advice

On the one hand, finding the balance of economic benefits, environmental release and health risks based on the empirical research. Firstly, adding external constraints such as capital cost, time cost, and human cost that enterprises need to invest for environmental release control and risk prevention, corresponding to each measure in the target case study. Secondly, constructing a multi-objective decision model based on greedy algorithm to find the local optimal solution under the above constraints of different cost inputs and risk levels, and establish a set of risk control and input strategies. For example, if an enterprise wants to reduce the probability of workers' cancer to < 3 per 100,000, the total amount of risk prevention funds to be invested and the investment strategy can be obtained by the decision model under the condition that the number of workers and the duration of work are determined.

On the other hand, the study helps to facilitate the establishment of a long-term occupational health risk management system and the introduction of more accurate environmental release and worker occupational health risk assessment criteria for the production of PC components, as well as more accurate occupational health risk monitoring and early warning by the government. Occupational health risk long-term management system construction, risk monitoring and early warning aims to establish a set of long-term monitoring and early warning management system based on the above research results, specifically including: (1) Integrate the multi-objective model with the simulation model, combine simulation experiments with parallel experiments, and establish environmental release and worker occupational health risk evaluation criteria for PC component production for different products (wall/floor slabs, stairs, shield pieces, etc.) and different types of work (reinforcement tying, cement pouring, component maintenance workers, etc.), respectively. (2) Determine the number, location and scope of environmental release monitoring points according to the standards, set the concentration/intensity monitoring thresholds for different categories of emissions, and establish a multi-level risk warning system for workers' carcinogenesis and chronic diseases. (3) Compare the monitoring data of several PC component plants, summarize the characteristics of environmental release and workers' health risks, further optimize the monitoring program of monitoring points, thresholds and types of work, and form evaluation standards with high timeliness and universality, and promote and apply them in the whole industry.

## Conclusion

The occupational health risks of construction industry workers have been much higher than the social average. With the growing scale of prefabricated buildings, the work originally required to be done on site is shifting to the front end, and the health risks of on-site construction workers are shifting to industrial workers in prefabricate concrete component plants.

Based on the intersection of engineering, environmental, and health management, this study tries to solve the root problems behind occupational health risks from risk identification, risk assessment, and risk control, the three processes such as the lack of measurement, assessment, and prevention standards. Through mapping physical entities to virtual simulation, feeding back the quantification and optimization results to practical applications, and then expanding individual projects to the whole industry, this study provides new ideas for theoretical research and innovative practice of HSE management and risk management in the construction industry. Firstly, the logical framework of occupational health risk is formed using the concept of life cycle risk management, which provides a new paradigm for the research in the field of occupational health risk management. Secondly, identifying the characteristics of the source of emissions and analyzing the diffusion propagation law and diffusion mechanism to understand the whole path process of environmental release. Afterwards, clarifying the human intake mechanism combined with the personal exposure assessment method that considers uncertainty factors. Finally, the simulation model of occupational health risk assessment for the whole process workers is formed, and finally it is applied to real cases to form a complete set of long-term management model of occupational health risks caused by environmental release.

A reasonable risk assessment simulation model and a long-term management model are proposed in this study. In the future, more empirical researches need to be verified in the model to improve the measurement accuracy, refine the human exposure assessment method, and adjust the simulation parameters, and so as to help decision makers to formulate occupational health risk policy. Furthermore, the production of PC components is only one phase of the assembled building industry. Transport, construction and other phases also include a large number of workers whose health risks need to be considered in future studies.

## Data availability statement

The original contributions presented in the study are included in the article/supplementary material, further inquiries can be directed to the corresponding author/s.

## Author contributions

PC led the overall study. HZ, ZD, and XJ conducted the analysis. HZ, ZD, XJ, PZ, and SZ drafted the manuscript with input from the other authors. All authors contributed to the concept and design of the study, the interpretation of data, revision of the manuscript for important intellectual content, and have read and approved the final version of the manuscript.

## Funding

This research was funded by Jiangsu Natural Science Fund (BK20200782) and Innovation and entrepreneurship training program for college students in Jiangsu Province (202110298026Z).

## Conflict of interest

The authors declare that the research was conducted in the absence of any commercial or financial relationships that could be construed as a potential conflict of interest.

## Publisher's note

All claims expressed in this article are solely those of the authors and do not necessarily represent those of their affiliated organizations, or those of the publisher, the editors and the reviewers. Any product that may be evaluated in this article, or claim that may be made by its manufacturer, is not guaranteed or endorsed by the publisher.
